# Prevalence and incidence rate of lower-extremity tendinopathies in a Danish general practice: a registry-based study

**DOI:** 10.1186/s12891-019-2629-6

**Published:** 2019-05-22

**Authors:** Henrik Riel, Cassandra Frydendal Lindstrøm, Michael Skovdal Rathleff, Martin Bach Jensen, Jens Lykkegaard Olesen

**Affiliations:** 1 Center for General Practice at Aalborg University, Fyrkildevej 7, 9220 Aalborg East, Denmark; 2 Department of Health Science and Technology, Faculty of Medicine, Aalborg University, Niels Jernes Vej 10, 9220 Aalborg East, Denmark; 3 Center for Sensory-Motor Interaction (SMI), Department of Health Science and Technology, Faculty of Medicine, Aalborg University, Fredrik Bajers Vej 7D, 9220 Aalborg East, Denmark

**Keywords:** Tendinopathy, Epidemiology, Prevalence, Incidence rate, General practice

## Abstract

**Background:**

Tendinopathies of the lower extremity (e.g. Achilles, patellar, and plantar heel pain) are common in both general and sporting populations. However, the prevalence and incidence in Danish general practice is unknown. The aim was to determine the prevalence and incidence rate of lower-extremity tendinopathies in a Danish general practice.

**Methods:**

In this registry-based study, we extracted data from the electronic patient files of all patients in a single Danish general practice. The practice included 8836 patients. We searched ICPC-2 codes to identify patients with either of the following lower-extremity tendinopathies: plantar heel pain; Achilles tendinopathy; patellar tendinopathy; greater trochanteric pain syndrome or adductor tendinopathy. We defined an incident and prevalent case as a patient with a consultation because of tendinopathy in 2016 only. A prevalent, but not incident case was a patient with consultations in both 2015 and 2016. Incidence and prevalence were expressed as the number of patients with a tendinopathy per 1000 registered patients.

**Results:**

The prevalence and incidence rate were 16.6 and 7.9 per 1000 registered patients, respectively. Plantar heel pain was the most prevalent tendinopathy and accounted for 39% of lower-extremity tendinopathies. Patients with tendinopathies were significantly older than all registered patients (46.0 years (95%CI: 43.3;48.7) versus 38.8 years (95%CI: 38.4;39.3), respectively).

**Conclusions:**

Lower-extremity tendinopathies, especially plantar heel pain, had a high prevalence and incidence rate in a Danish general practice. In a typical general practice with 5000 patients, general practitioners should expect to see more than 80 patients with a lower-extremity tendinopathy every year.

## Background

Tendinopathies are characterised by clinical symptoms that may consist of activity-related pain, dysfunction, tenderness, swelling and increased stiffness of a tendon [1–4]. Tendinopathy can occur in all tendons, but are commonly observed in larger tendons such as the Achilles and patellar [5]. While several intrinsic and extrinsic factors have been suggested to predispose to injury, the precise pathogenesis remains unknown [2, 3]. Tendinopathy often impacts the patient’s daily life in multiple biopsychosocial ways such as employment, mental health and quality of life on both a personal and a professional level [6]. Tendinopathy has been related to reduced work ability and decreased work productivity, particularly for physically demanding work [7].

General practitioners are important in the management of tendinopathies as they are usually the first point of contact and first to deliver treatment to patients. It is essential to know the prevalence and incidence rate of lower-extremity tendinopathies to shed light on the extent of their burden in general practice. This may also inform the planning of future research on tendinopathies by helping to establish the feasibility of recruiting from general practice for clinical studies and, moreover, depending on the distribution of the various tendinopathies, the need for prioritising between the different tendinopathies. The prevalence and incidence rate of lower-extremity tendinopathies in a Dutch general practice population have previously been investigated, but never in a Danish general practice setting [8].

The aim of this study is to determine the prevalence and incidence rate of lower-extremity tendinopathies in a Danish general practice.

## Methods

### Study design

To investigate the prevalence and incidence rate of lower-extremity tendinopathies in a Danish general practice, we conducted an observational registry-based study. Reporting of the study follows the RECORD Statement Guidelines (The REporting of studies Conducted using Observational Routinely collected health Data) [9].

### Setting

Data were extracted at a general practice clinic (Aalborg, Denmark) by CFL between October 23rd 2017 and December 6th 2017. CFL was trained in extracting data by an experienced medical doctor (MBJ). Three full-time general practitioners, seven nurses, and changing residence doctors worked at the clinic at the time of data extraction. In 2016, 8836 patients consulted the clinic.

### Study population selection

Using International Classification of Primary Care 2 codes (ICPC-2), we searched the electronic patient files to identify diagnoses encompassing the following lower-extremity tendinopathies: plantar heel pain [10], Achilles tendinopathy (midsubstance and insertional), patellar tendinopathy, greater trochanteric pain syndrome (GTPS) and adductor tendinopathy. ICPC-2 is a classification system for general practice and primary care [11]. An ICPC-2 code often represents a symptom, disease or a combination of these. There are few tendinopathy-specific ICPC-2 codes (e.g. L93 = lateral elbow tendinopathy). Therefore, the unspecific ICPC-2 code L87 (Bursitis/tendinitis/synovitis not otherwise specified) is commonly given to patients in general practice. On rare occasions, the ICPC-2 code L78 (sprain/strain of knee) is given in the case of patellar tendinopathy (Jumper’s Knee). Patients included in the present study were identified by searching for ICPC-2 codes L87 and L78. To determine eligibility and specific tendinopathy classification, CFL studied the free-text section of patients’ files that had been assigned with either of these codes. After the initial evaluation of the electronic patient files, JLO and HR independently reviewed them and had the final decision over classification. To allow for comparisons of age and sex between patients with lower-extremity tendinopathies and the entire GP population, we searched for all patients who had a consultation in 2016 and extracted their sex and age.

The inclusion criteria were decided for each tendinopathy based on a consensus among the authors before data extraction (Table 1). Patients diagnosed with more than one type of lower-extremity tendinopathy were only included once in the analyses with the diagnosis they had first consulted their GP with. Moreover, patients with bilateral tendinopathy only counted as one case. For each patient, data regarding age, sex, type of tendinopathy and initial date the patient received the diagnosis were extracted.

**Table 1:** inclusion criteria for each tendinopathy

Tendinopathy	Description in patient file
Plantar heel pain	- Plantar fasciopathy/Fasciitis plantaris - Heel spur - Calcar calcanei - Contractura aponeuroseos plantaris
Achilles tendinopathy	- Achilles tendinopathy/tendinitis - Tendinitis calcanei
Patellar tendinopathy	- Patellar tendinopathy/ tendinitis - Jumper’s Knee
Greater trochanteric pain syndrome	- Greater trochanteric pain syndrome - Trochanteric bursitis - Tendinitis trochanterica
Adductor tendinopathy	- Adductor tendinopathy - Tendinitis of the hip adductors

### Data extracted from records

Electronic patient files dated from January 1st 2015 to December 31st 2016 were searched. Prevalent and incident cases were defined based on when the diagnosis had been entered into the patient record. We defined an incident and prevalent case as a patient with a consultation because of tendinopathy in 2016 only. A prevalent, but not incident case was a patient with consultations in both 2015 and 2016. This definition is similar to that of previous research [8].

### Statistical analyses

To calculate the prevalence and incidence rates per 1000 registered patients, we used the following equation: number of prevalent or incident cases/number of registered patients*1000. Regardless of the number of consultations in 2016, each patient was only counted as one incident case. To investigate a potential age difference between the two groups, we calculated 95% confidence intervals. If the confidence intervals did not overlap, we would conclude a between-group difference [12]. Furthermore, to explore potential differences in age and sex distribution between patients with and without tendinopathies, we sub-grouped patients according to age and performed χ^2^ tests with an alpha level of 5%. Sub-grouping was in accordance with a similar previous study [8]. Data regarding sex and age of patients with tendinopathies could not be isolated from the entire practice population and are present in both groups. Therefore, we used a correction factor to adjust χ^2^ statistic and confidence intervals for overlapping cases. [13] Data were analysed with Microsoft Excel (Microsoft Corporation, Washington, USA).

## Results

The search from patient records retrieved 421 patient files including a total of 632 visits. Of these, 274 patient files were excluded due to irrelevant diagnoses. 147 prevalent cases (consultations in 2015 and/or 2016) of lower-extremity tendinopathies were identified, with 70 incident cases identified (consultations in 2016 only). This led to a prevalence of 16.6 cases per 1000 registered patients and an incidence rate of 7.9 cases per 1000 registered patients. Plantar heel pain (57 cases) and Achilles tendinopathy (46 cases) were the most prevalent tendinopathies (Table 2). We found eight cases where the free-text section of the patient files indicated a lower-extremity tendinopathy, but we could not determine the specific tendinopathy.

**Table 2:** Distribution Of Tendinopathies

	Prevalent cases (%)	Prevalence (/1000 registered patients)	Incident cases (%)	Incidence (/1000 registered patients)
Plantar heel pain	57 (39)	6.5	33 (47)	3.7
Achilles tendinopathy	46 (31)	5.2	15 (21)	1.7
Patellar tendinopathy	10 (7)	1.1	4 (6)	0.5
Greater trochanteric pain syndrome	26 (18)	2.9	14 (20)	1.6
Adductor tendinopathy	0 (0)	0.0	0 (0)	0.0
Unspecified	8 (5)	0.9	4 (6)	0.5

Sex was not registered in 61 patient files of the total practice population but was registered in all of the files of the patients with tendinopathies. Sex distribution and mean age of patients are presented in Table 3 specifically for each tendinopathy. In both patients with and without tendinopathies there were significantly more women (*P* = 0.004 and *P* < 0.001, respectively) but there was no difference in the distributions between these groups. The mean age of patients with tendinopathies was significantly higher than of those without. The age distribution was also significantly different between the two groups as there was a higher proportion of patients between the ages from 0 to 17 among the general practice population compared to patients with tendinopathies (*P* < 0.001) and a higher proportion of patients with tendinopathies between the ages from 45 to 64 when compared to the proportion in the general practice population (*P* < 0.001) (Table 4).

**Table 3:** Patient characteristics per tendinopathy

	Mean age (SD)	Sex Female (%)/Male (%)
Plantar heel pain	45.1 (16.5)	35 (61)/22 (39)
Achilles tendinopathy	49.0 (17.1)	21 (46)/25 (54)
Patellar tendinopathy	32.5 (10.7)	5 (50)/5 (50)
Greater trochanteric pain syndrome	50.8 (15.2)	19 (73)/7 (27)
Unspecified	36.3 (17.1)	6 (75)/2 (25)

**Table 4:** Distribution of sex and age

	Patients with tendinopathies	Practice population
Sex		
Male	41.5% (34.0 to 49.5)	47.8% (46.8 to 48.9)
Female	58.5% (50.5 to 66.1)	52.2% (51.1 to 53.2)
Age		
Mean years^a^	46.0 (43.3 to 48.7)	38.8 (38.4 to 39.3)
Age Groups		
0 to 17^a^	5.4% (2.8 to 10.3)	15.9% (15.2 to 16.7)
18 to 44	39.5% (32.0 to 47.4)	45.5% (44.5 to 46.6)
45 to 64^a^	36.7% (29.5 to 44.7)	21.9% (21.0 to 22.7)
65+	18.4% (13.0 to 24.9)	16.7% (16.0 to 17.5)

Data presented as proportion (95% CI) unless otherwise stated

^a^Statistically significant between-group differences at an alpha-level of 5% based on either overlapping confidence intervals or χ^2^ tests

## Discussion

### Key results

This was the first study to investigate the prevalence and incidence rate of lower-extremity tendinopathies in a Danish general practice population. We found a prevalence of 16.6 per 1000 registered patients and an incidence rate of 7.9 per 1000 registered patients. Hence, in a typical general practice with 5000 patients, the general practitioners should expect to see more than 80 patients with a lower-extremity tendinopathy every year. Plantar heel pain was the most prevalent followed by Achilles tendinopathy whereas there was not a single case of adductor tendinopathy. Patients with tendinopathies were generally older than the general practice population.

### Interpretation of findings

Our findings support previous studies of lower-extremity tendinopathies in Dutch general practice populations [8, 14, 15]. A study from 2011 found an incidence rate of 1.85 per 1000 registered patients for midportion Achilles tendinopathy whereas we found an incidence rate of 1.7 in the present study. Lievense et al. found an incidence rate of GTPS as primary pain of 1.8 per 1000 patients in the Dutch population while we found an incidence rate of 1.6 [15]. Thus, incidence rates of lower-extremity tendinopathies may be similar in the Netherlands and Denmark. This may even be generalised to other countries with similar healthcare systems, geographical location and socio-economic status as the socio-economic status may influence health [16, 17].

A more recent study from 2016 of a Dutch general practice population investigated the prevalence and incidence of the same lower-extremity tendinopathies that we included, but found slightly fewer prevalent cases than we did (11.8/1000 patients versus 16.6/1000 patients, respectively) [8]. Interestingly, the distribution of the tendinopathies found in that study was different from the distribution of the present study. GTPS was almost twice as prevalent as plantar heel pain in the study by Albers et al. whereas we found plantar heel pain to be more than twice as prevalent as GTPS. This discrepancy may be explained by how the ICPC-2 codes are used in practice and a low validity of coding as the demographic characteristics of the two general practice populations appear very similar in terms of both age and sex. Potentially, a large proportion of GTPS cases of the Albers et al. study may have been derived from their search of ICPC-2 code L13 which is a code used for general symptoms or complaints of the hip [8]. Another difference between the two studies’ findings is that we did not retrieve a single case of adductor tendinopathy whereas Albers et al. found 13 prevalent cases corresponding to approximately 10% of all cases [8]. The reason for this difference is unknown, but may potentially be caused by a misclassification by the Danish GPs as the two populations were otherwise very similar. This may form a basis for exploring GPs’ knowledge about adductor tendinopathy in the future.

In accordance with Albers et al., we found that the mean age of patients with lower-extremity tendinopathies was significantly higher than that of the general practice population [8]. The largest difference in age distribution between the two populations was seen between the ages of 45 to 64. Age may increase the prevalence and risk of lower-extremity tendinopathies. This is supported by previously reported age-related changes which may predispose to tendon injuries. Age-related changes can result in both decreased cell regeneration and reduced ability to tolerate high loads in older individuals [18]. Thus, older tendons are generally more susceptible to injury. Despite increased susceptibility to tendon pain with age, we did not observe any proportional difference between the populations among patients aged + 65. This could either be explained by an age-related decrease in sports participation as tendinopathies such as Achilles and patellar are commonly seen in sporting populations or by a decrease in work-related loads due to retirement [2, 19].

A review of the pathophysiology of tendinopathies suggested that females are at a higher risk of developing tendinopathies which is in line with our findings as 58.5% of those with tendinopathies were women [20]. This may also be associated with age as a decrease in tendon collagen synthesis has been found in postmenopausal women [21]. However, the proportion of women:men may be different from one tendinopathy to another. E.g. the majority of patients with tendinopathies were women, but there was an equal distribution between the sexes among those with patellar tendinopathy (5/5). In fact, in the literature, males have been found to have a higher risk of developing patellar tendinopathy compared to women [22]. Hence, we cannot necessarily generalise which of the sexes is more prone to developing tendinopathies from one lower-extremity tendinopathy to them all.

### Clinical and research implications

Studies of general practitioners’ confidence in managing obese patients [23], elderly patients [24] and patients with dementia [25] have been made in the past, however, to our knowledge, never their confidence in managing patients with tendinopathies. In light of the large extent of lower-extremity tendinopathies seen in general practice, this would be highly relevant to investigate in the future. The frequency by which general practitioners see patients with tendinopathies suggests feasibility of recruiting patients for clinical trials from general practice. As recruitment for trials is often a challenge, future research could focus on how to best recruit from general practice to benefit both patients, practitioners and researchers alike.

### Limitations and strengths

Our study has some limitations. Due to the retrospective nature of the study, it is not possible to confirm the diagnoses and we rely solely on ICPC-2 codes and the general practitioners who assigned them. We only included a single general practice with three general practitioners and a number of temporary residence doctors. Therefore, results are more susceptible to be influenced by the evaluation of the individual practitioners and the potential misclassification compared with a clinic with even more general practitioners employed. It is likely that we underestimate the actual prevalence of tendinopathies because; 1) we only included two ICPC-2 codes and despite the fact that these should embrace all lower-extremity tendinopathies, it is possible that some cases have been given a wrong ICPC-2 code by the general practitioners, 2) we only included patients with consultations in 2015 and 2016 but due to the chronic nature of several tendinopathies, there may have been patients who were diagnosed with a tendinopathy before 2015 and still had active symptoms in 2016 but had stopped visiting the general practice for the condition, and 3) in general, only one in three with musculoskeletal complaints will consult their general practitioner and specifically for plantar heel pain, the number is only three in four. [26, 27] Therefore, it is highly probable that the actual magnitude of these conditions is larger than what can be estimated from this present study.

The comparability of this study’s findings with those of similar studies from the Netherlands is one of its strength. This emphasises the generalisability of the results. This is further supported by the similarities in demographic characteristics of the practice population and those of the practice population in Albers et al. [8] Another strength is that three of the authors evaluated the free-text sections of the electronic patient files to minimise the risk of a wrong patient classification.

## Conclusions

Lower-extremity tendinopathies were found to have a prevalence of 16.6 per 1000 registered patients and an incidence rate of 7.9 per 1000 registered patients in a Danish general practice. Plantar heel pain was the most prevalent tendinopathy and accounted for 39% of all lower-extremity tendinopathies. In a typical general practice with 5000 patients, the general practitioners should expect to see more than 80 patients with a lower-extremity tendinopathy every year.

## Abbreviations

GTPS: Greater trochanteric pain syndrome
ICPC-2: International classification of primary care 2 codes
